# Honey bee Royalactin unlocks conserved pluripotency pathway in mammals

**DOI:** 10.1038/s41467-018-06256-4

**Published:** 2018-12-04

**Authors:** Derrick C. Wan, Stefanie L. Morgan, Andrew L. Spencley, Natasha Mariano, Erin Y. Chang, Gautam Shankar, Yunhai Luo, Ted H. Li, Dana Huh, Star K. Huynh, Jasmine M. Garcia, Cole M. Dovey, Jennifer Lumb, Ling Liu, Katharine V. Brown, Abel Bermudez, Richard Luong, Hong Zeng, Victoria L. Mascetti, Sharon J. Pitteri, Jordon Wang, Hua Tu, Marco Quarta, Vittorio Sebastiano, Roel Nusse, Thomas A. Rando, Jan E. Carette, J. Fernando Bazan, Kevin C. Wang

**Affiliations:** 1 Department of Dermatology, Program in Epithelial Biology, Stanford University School of Medicine, Stanford, CA 94305 USA; 2 Hagey Laboratory for Pediatric Regenerative Medicine, Stanford University School of Medicine, Stanford, CA 94305 USA; 3 Program in Cancer Biology, Stanford University School of Medicine, Stanford, CA 94305 USA; 4 Department of Microbiology and Immunology, Stanford University School of Medicine, Stanford, CA 94305 USA; 5 The Glenn Laboratories for the Biology of Aging and Department of Neurology and Neurological Sciences, Stanford University School of Medicine, Stanford, CA 94305 USA; 6 Department of Developmental Biology, Howard Hughes Medical Institute, Stanford Institute for Stem Cell Biology and Regenerative Medicine, Stanford University School of Medicine, Stanford, CA 94305 USA; 7 Canary Center for Cancer Early Detection, Department of Radiology, Stanford University School of Medicine, Palo Alto, CA 94304 USA; 8 Department of Comparative Medicine, Stanford University School of Medicine, Stanford, CA 94305 USA; 9 Institute for Stem Cell Biology and Regenerative Medicine, Stanford University School of Medicine, Stanford, CA 94305 USA; 10 LakePharma, Inc., Belmont, CA 94002 USA; 11 R&D Systems, Inc, Minneapolis, MN 55413 USA; 12 Veterans Affairs Palo Alto Healthcare System, Palo Alto, CA 94304 USA

## Abstract

Royal jelly is the queen-maker for the honey bee *Apis mellifera*, and has cross-species effects on longevity, fertility, and regeneration in mammals. Despite this knowledge, how royal jelly or its components exert their myriad effects has remained poorly understood. Using mouse embryonic stem cells as a platform, here we report that through its major protein component Royalactin, royal jelly can maintain pluripotency by activating a ground-state pluripotency-like gene network. We further identify Regina, a mammalian structural analog of Royalactin that also induces a naive-like state in mouse embryonic stem cells. This reveals an important innate program for stem cell self-renewal with broad implications in understanding the molecular regulation of stem cell fate across species.

## Abstract (web summary)

Royal jelly is the queen-maker for the honey bee that also has effects on longevity, fertility, and regeneration in mammals. Here the authors provide evidence that its major protein component Royalactin, and the mammalian structural analog Regina, maintain pluripotency in mouse ESCs by activating a ground-state pluripotency-like gene network.

## Introduction

The regulation of self-renewal and differentiation potential in mouse embryonic stem cells (mESCs) occurs through complex transcriptional networks orchestrated by conserved transcription factors^1,2^. Although differences exist in the specific signaling pathways that control self-renewal and lineage development, culture conditions that allow for derivation and maintenance of stem cells have been identified^3–8^. In particular, use of two small molecule inhibitors targeting MAPK/ERK Kinase (Mek) and glycogen synthase kinase-3 (GSK3) in addition to Leukemia inhibitory factor (LIF) in serum-free media permitted derivation of germline-competent ESCs that resemble the mature mouse inner cell mass (ICM)^9^. However, recent findings suggest that prolonged Mek1/2 suppression may have detrimental effects on the epigenetic and genetic integrity of mESCs, effectively limiting their developmental potential^10,11^. As such, additional methods of maintaining mESCs in an ICM state are required.

Though best-known as an epigenetic driver of queen development in *A. mellifera*^12^, the functional component of royal jelly, Major Royal Jelly Protein 1 (MRJP1, also known as Royalactin), has been shown to modulate biological function in a broad range of species^12–15^. Indeed, conservation of increased growth stimulation and cellular proliferation phenotypes in response to MRJP1 has been observed in murine hepatocytes^16–18^. While this indicates a functionally important role for this royal jelly protein in regulating cell state and fate, the full scope of its effects has not yet been well characterized^19^.

In this study, we identify Royalactin as a potent activator of a pluripotency gene network through modulation of chromatin accessibility, that maintains mESC self-renewal in the absence of LIF. Royalactin cultured cells also occupy a more naive ground state capable of generating chimeric animals with germline transmission. Finally, we identify a mammalian structural analog of Royalactin possessing similar functional capacity, uncovering a molecular conservation that supports distinct evolutionary processes.

## Results

### Royalactin maintains mESC self-renewal and pluripotency

**Fig. 1:**
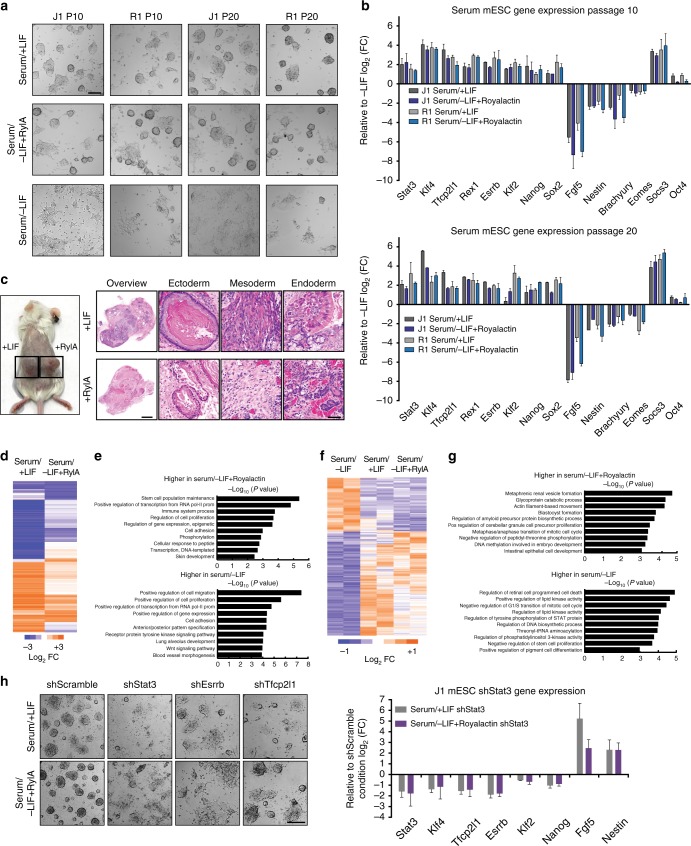
Royalactin maintains stemness in murine embryonic stem cells. **a** Representative images of J1 and R1 mESCs cultured in serum/+LIF, serum/−LIF, or serum/−LIF + Royalactin for 10 and 20 passages. After LIF withdrawal, mESCs rapidly differentiated, whereas cells cultured with Royalactin supported self-renewal with negligible differentiation. Scale bar, 200 μm. **b** Quantitative expression of pluripotency and differentiation-associated genes from **a**. Data are means ± SD (*n* = 2). **c** Mice bearing mESC-derived teratomas from J1 mESCs cultured three passages in +LIF and −LIF + Royalactin demonstrated retained pluripotency, and on high magnification (400×) produced differentiated ectodermal, mesodermal, and endodermal tissues. Scale bar, 80 μm. **d** RNA-seq log2-fold change values in transcript level of all genes in serum/+LIF or serum/–LIF + Royalactin J1 mESCs (passage 10) relative to serum/–LIF. **e** GO term analysis of differentially expressed genes from **d**. **f** ATAC-seq activity in J1 mESCs at passage 10. Each column is a sample, each row is an element. Samples and elements are organized by unsupervised k-means clustering. **g** GO term analysis of differentially accessible regions from **f**. **h** Representative images of Stat3, Esrrb, and Tfcp2l1 knockdown in J1 mESCs with serum/+LIF and serum/−LIF + Royalactin conditions and qPCR analysis of pluripotency and differentiation-associated genes from the same cells. Data are means ± SD (*n* = 2). Scale bar, 200 μm. RylA Royalactin

As mESCs provide a powerful model with which to study cellular regulation through pluripotency and differentiation programs, we first employed this model to dissect Royalactin’s mechanisms of action on mammalian cells. Upon LIF withdrawal (serum/–LIF), mESCs previously cultured in serum/+LIF media readily demonstrated a differentiated morphology as expected (Fig. 1a). Surprisingly, addition of Royalactin in the absence of LIF (serum/–LIF + Royalactin) for multiple passages resulted in dose-dependent formation of undifferentiated colonies that demonstrated similar morphology to those grown in the presence of LIF (Fig. 1a, Supplementary Figure 1a). Gene expression profiles of the cells grown in serum/–LIF + Royalactin further demonstrated a high degree of similarity relative to those cultured in the presence of LIF (Fig. 1b, Supplementary Figure 1b).

Having observed a robust stemness-maintenance effect of Royalactin in vitro, we next examined whether mESCs treated with Royalactin in serum/–LIF retain embryonic identity and developmental potential in vivo. Indeed, mESCs grown in serum/–LIF + Royalactin were grafted subcutaneously and gave rise within 6 weeks to large multi-differentiated teratomas (Fig. 1c). Collectively, these results indicate that Royalactin can functionally maintain self-renewal and pluripotency in mESCs.

### Royalactin modulates chromatin and pluripotent networks

In the interest of understanding Royalactin’s effects on the transcriptome, RNA-seq analyses of serum/+LIF, serum/–LIF, and serum/–LIF + Royalactin cells were conducted. These analyses revealed a strong enrichment for canonical pluripotency genes and a suppression of lineage-specific genes in serum/–LIF + Royalactin cells at levels similar to those in mESCs cultured in serum/+ LIF (Fig. 1d). Gene Ontology (GO) term analysis of all genes differentially expressed in the presence of Royalactin revealed strong enrichment for genes involved with proliferation and stemness in the upregulated gene set, and an overrepresentation of developmental processes in the downregulated gene set (Fig. 1e). Similarly, analysis of chromatin accessibility using the assay for transposase-accessible chromatin using sequencing (ATAC-seq)^20^ of mESCs cultured in serum/–LIF + Royalactin and serum/+LIF conditions revealed similar patterns of increases in ATAC-seq signal relative to mESCs grown in serum/–LIF conditions (Fig. 1f), specifically at promoters (TSS; 14234 total peaks with 7373 gaining accessibility and 6861 losing accessibility; Supplementary Figure 1c), traditional enhancers (TE; 5356 total peaks with 2571 gaining accessibility and 2785 losing accessibility; Supplementary Figure 1d), and super enhancer regions (SEs; 127 of 231 elements gaining accessibility; Supplementary Figure 1e). As expected, high correlation was observed between ATAC-seq changes and RNA-seq experiments (Supplementary Figure 1c–e), with functional annotation revealing that the ATAC-seq changes were located near genes associated with pluripotency, metabolism, and differentiation (Fig. 1g). Furthermore, motif enrichment analysis revealed that TFs such as KLF5, KLF4, and SOX2 bound at high frequency to the Royalactin-upregulated SE constituents (Supplementary Figure 1f). Collectively, this suggested that regulatory regions are sensitive to Royalactin culture conditions and cause subsequent changes in gene expression.

In order to gain a molecular understanding of patterns of gene expression and identify candidate regulators of pluripotency in response to Royalactin, a transcriptional network analysis was performed that identified *Stat3*, *Tfcp2l1*, *Esrrb*, and *Nanog* as the most significant nodes (Supplementary Figure 1g). Subsequent experimentation revealed a dose-dependent effect for Royalactin in stimulating phospho-*Stat3* activation concomitant with other pluripotency factors (Supplementary Figure 1h), which was sustained after 10 and 20 passages (Supplementary Figure 1i). In addition, knockdown of these transcription factors greatly diminished the mESC response to Royalactin, with the most significant effects being observed following Stat3 knockdown (Fig. 1h). As these findings suggested that Royalactin triggers activation of a Stat3-driven LIF-independent pathway on mESC self-renewal, further analysis of gene expression profiles from serum/+LIF and serum/–LIF + Royalactin mESCs were compared to identify 519 genes that are specifically activated in response to Royalactin (Supplementary Figure 1j). GO term analysis showed enrichment of metabolic and biosynthetic processes (Supplementary Figure 1k), reminiscent of mESCs cultured without serum in the presence of inhibitors targeting mitogen-activated protein kinase kinase and GSK3 (2i)^21^. Global expression profiles from serum/–LIF + Royalactin and 2i-cultured cells also clustered together by principal component analysis away from serum/+LIF-cultured cells (Supplementary Figure 1l). GO term enrichment analysis found that genes involved in basic metabolism, transcription, and development were responsible for this separation (Supplementary Figure 1m). Collectively, this suggested that Royalactin may be driving ground-state pluripotency in mESCs.

### Royalactin treated mESCs mimic ground-state pluripotency

**Fig. 2:**
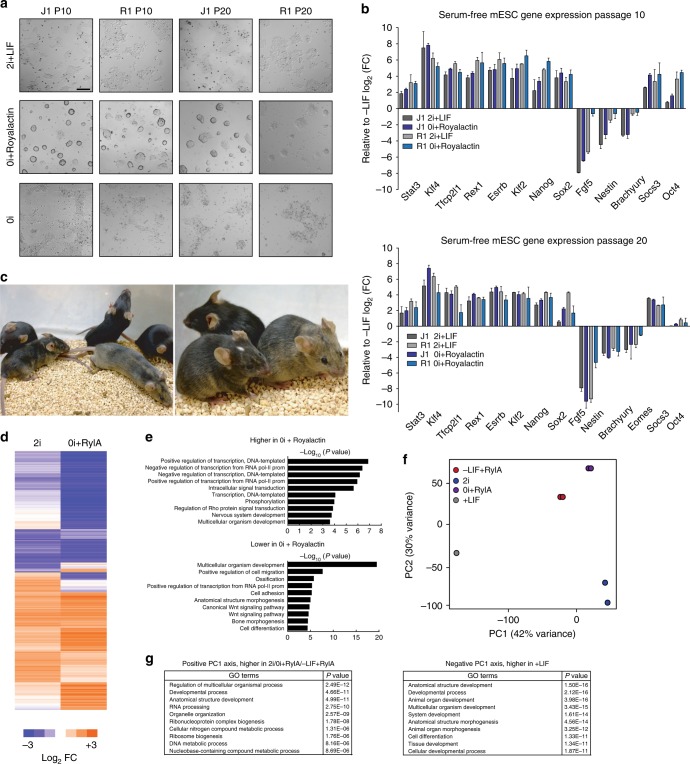
Royalactin drives a ground-state-like pluripotency state in mESCs. **a** Representative images of J1 and R1 mESCs cultured in serum-free media in the presence (2i + LIF) or absence (0i) of MAPKKi, GSK3i, and LIF for 10 and 20 passages. mESCs rapidly differentiated in 0i, whereas cells cultured with Royalactin (0i + Royalactin) supported self-renewal with negligible differentiation. Scale bar, 200 μm. **b** Quantitative expression of pluripotency and differentiation-associated genes from **a**. Data are means ± SD (*n* = 2). **c** Chimeras with germline transmission formed by CGR 8.8 mESCs treated for ten passages with 0i + Royalactin. **d** RNA-seq log_2_ fold change values in transcript level of all genes in 2i or 0i + Royalactin (0i + RylA) J1 mESCs (passage 10) relative to 0i. **e** GO term analysis of the differentially expressed genes from **d**. **f** J1 mESCs cultured in serum/+LIF, serum/−LIF + Royalactin, 2i + LIF, and 0i + Royalactin for ten passages are projected onto the first two principal components. All genes with mean normalized read counts larger than 10 were considered for principal component analysis (PCA). **g** Distribution of genes contributing to principal component 1 (PC1) in **f**, and GO enrichment analysis of genes most strongly contributing to PC1 separation. RylA Royalactin

We next sought to test the hypothesis that Royalactin was driving a ground-state-like pluripotency state in mESCs. As expected, mESCs cultured in 2i + LIF media maintained pluripotency and sustained expression of a Rex1 GFP pluripotency marker^22^, while those in 2i base media without inhibitors (0i) readily differentiated (Fig. 2a, b, Supplementary Figure 2). Remarkably, addition of Royalactin in the absence of inhibitors (0i + Royalactin) for multiple passages maintained undifferentiated GFP positive colonies with similar gene expression profiles to 2i + LIF cultured cells (Fig. 2a, b, Supplementary Figure 2). In addition, injection of 0i + Royalactin cultured cells into mouse blastocysts generated chimeric animals with germline transmission, highlighting the robust effects of this protein in vivo (Fig. 2c, Supplementary Table 1).

RNA-seq and GO-term analyses further demonstrated marked similarities in enriched genes between 2i + LIF and 0i + Royalactin cells (Fig. 2d, e), and global expression profiles from 2i + LIF, 0i + Royalactin, serum/+LIF, serum/–LIF + Royalactin, and serum/–LIF cells demonstrated a clear clustering by principal component analysis of 0i + Royalactin cells nearer those cultured in 2i + LIF than those cultured in serum/+LIF (Fig. 2f). GO term enrichment analysis found that genes involved in basic metabolism, transcription, and development were responsible for this separation (Fig. 2g). These data suggest that Royalactin treatment is accompanied by a profound metabolic reprogramming resembling the 2i + LIF state, mimicking the environment of the mature mouse ICM.

### Identification of Royalactin mammalian analog

**Fig. 3:**
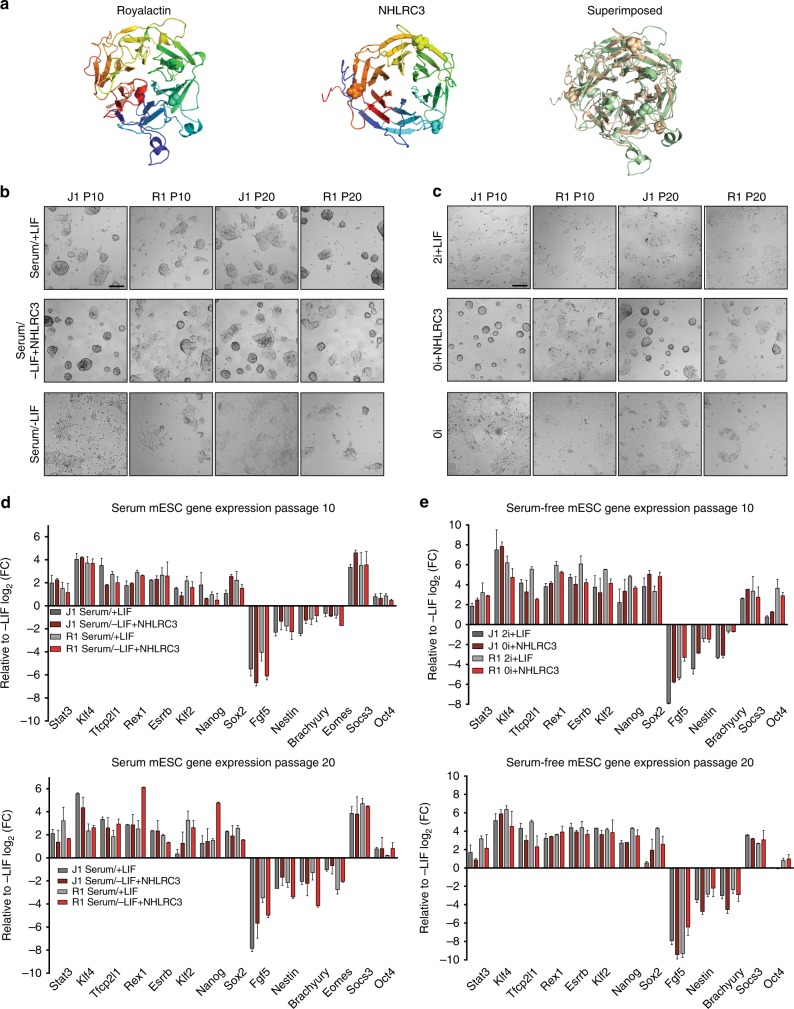
The mammalian structural analog of Royalactin maintains naive and ground-state pluripotency in mouse embryonic stem cells. **a** Computational modeling predicts the structure of Royalactin (left), allowing for identification of a highly structurally analogous protein, NHLRC3 (center). Superimposition of these models (right) demonstrates striking similarity between them. **b** Representative images of J1 and R1 mESCs cultured in serum/+LIF, serum/–LIF, or serum/−LIF + NHLRC3 for 10 and 20 passages. After LIF withdrawal, mESCs rapidly differentiated, whereas cells cultured with NHLRC3 supported self-renewal with negligible differentiation. Scale bar, 200 μm. **c** Representative images of J1 and R1 mESCs cultured in serum-free media in presence (2i + LIF) or absence (0i) of MAPKKi, GSK3i, and LIF for 10 and 20 passages. mESCs rapidly differentiated in 0i, whereas cells cultured with NHLRC3 (0i + NHLRC3) supported self-renewal with negligible differentiation. Scale bar, 200 μm. **d** Quantitative expression of pluripotency and differentiation-associated genes from **b**. Data are means ± SD (*n* = 2). **e** Quantitative expression of pluripotency and differentiation-associated genes from **c**. Data are means ± SD (*n* = 2)

We next wondered whether a homolog of Royalactin existed in mammals. Sensitive searches of sequence databases using iterative PSI-BLAST^23^, as well as aiming HHPRED sequence and structural profiles against the human and mouse proteomes^24^ did not reveal any Royalactin orthologs. However, the latter computational tool revealed that Royalactin is distantly related to an existing structure in the PDB database^25^, a secreted salivary gland protein (SGP) from the sand fly, *L. longipalpis* (PDB ID: 3Q6K)^26^. We then used this structure––a six-bladed β-propeller fold with no additional domains—as an accurate template for MODELLER^27^, yielding a high confidence model for the Royalactin fold (Fig. 3a). The resulting superposition of Royalactin and SGP sequences was then used to seed new and more precise HHPRED scans of the human proteome, in search of a possible structural and functional analog of the Royalactin/SGP β-propeller fold. Fitting the description of a secreted, single domain chain, with a predicted 6-bladed β-propeller architecture, only one protein, the provisionally named NHL Repeat Containing 3 (NHLRC3), arose as a potential candidate, with striking fold similarity to the Royalactin model (Fig. 3a). Although no known function of NHLRC3 has been identified to date, single-cell RNA-seq analyses of early mouse embryos revealed that it is expressed starting in E4.5 embryos, and that its expression increases steadily thereafter (Supplementary Figure 3a). To elucidate whether it served a functional purpose in stemness maintenance in mESCs similar to that observed with Royalactin, recombinant mouse NHLRC3 was added to mESC culture in the presence of serum/–LIF (serum/–LIF + NHLRC3) as well as 0i (0i + NHLRC3). As seen with Royalactin, NHLRC3 maintained mESCs in an undifferentiated state in both culture conditions for multiple passages (Fig. 3b, c, Supplementary Figure 3b, c), with expected changes in gene expression (Fig. 3d, e). Additionally, injection of 0i + NHLRC3 cultured cells into mouse blastocysts generated chimeric animals with germline transmission, highlighting the robust effects of this protein in vivo (Supplementary Figure 4, Supplementary Table 1). Thus, NHLRC3 appears to be a mammalian pluripotency maintenance factor, whose existence demonstrates a remarkable conservation of macromolecular structure and function. We renamed *NHLRC3* as *Regina* due to its conservation of functions with those of Royalactin and the queenmaker Royal Jelly.

In summary, our results demonstrate an unexpected capacity for Royalactin as a pluripotency factor that confers self-renewal and promotes emergence of the naive pluripotent gene regulatory network, and identify Regina as a factor that can promote ground-state pluripotency in mESCs. A better understanding of the interaction of Royalactin and Regina with genetic pathways and biochemical processes conserved in evolution, and how the different pluripotency networks function independently or synergistically to maintain stem cell self-renewal, will advance our efforts to better control stem cell fate, and provide a platform for dissection of the pluripotent state. Our findings and the discovery of Regina thus support the intriguing idea that profound molecular conservation underlies even the most evolutionarily novel traits. Future work in dissecting the mechanistic action of Royalactin in mammalian cells, including further characterization of its mammalian structural analog Regina, will shed new light on mammalian pluripotency and provide additional means to enhance optimal maintenance and derivation of ESCs for therapeutic application and regenerative medicine.

## Methods

### Embryonic stem cell culture

J1 and R1 mESCs (gift from Howard Y. Chang) were grown on 0.2% gelatin-coated (Sigma G6144) tissue culture plates. Cells were maintained for a minimum of ten passages in mESC serum medium containing KnockOut™ D-MEM (Life Technologies 10829), 15% HyClone™ fetal bovine serum (FBS; Thermo Scientific™ SH30396.03), 100 U/ml Penicillin-Streptomycin (P/S; Life Technologies 15140), 1% MEM non-essential amino acids (Life Technologies 11140), 1% GlutaMAX™ (Life Technologies 35050), and 0.1 mM 2-mercaptoethanol (Life Technologies 21985). Mouse leukemia inhibitory factor (LIF; Millipore ESG1107; 1000 U/mL), purified Royalactin (0.5 mg/mL), or purified NHLRC3 (0.125 mg/mL) were added to culture as indicated. Cells were passaged every 2 to 3 days using Trypsin-EDTA (0.25%; Life Technologies 25200) and seeded at 15,000 cells/cm^2^. Media and protein were changed daily.

For cultures under 2i conditions, J1, R1, and Rex1-GFP mESCs were grown on Poly-L-ornithine (Sigma-Aldrich)/Laminin (Fisher Scientific 23017–015) coated plates and CGR8.8 mESCs were grown on Matrigel (Corning 354277) plates coated according to manufacturer’s specifications. All cells were maintained a minimum of ten passages in serum-free media containing: 1:1 Neurobasal:DMEM-F12 base (Thermo Scientific 21103049), 1% Glutamax, 1% high insulin N2 (Thermo Scientific 17502001), 1% B27 supplement (Thermo Scientific 12587001), 0.1 mM 2-mercaptoethanol, 1% Penicillin-Streptomycin, 1% MEM non-essential amino acids, 1% sodium pyruvate (Thermo Scientific 11360070). Mouse leukemia inhibitory factor (LIF; Millipore ESG1107; 1000 U/mL), 1 μM PD0325901 (Selleckchem), 3 μM CHIR99021 (Selleckchem), purified Royalactin (0.5 mg/mL), or purified NHLRC3 (0.125 mg/mL) were added to cultures as indicated. Cells were passaged every 2 to 3 days using Accutase (Stemcell Technologies 07920) and seeded at 15,000 cells/cm^2^. Media and protein were changed daily.

### Production of recombinant Royalactin and NHLRC3

FLAG-MRJP1-His (Genbank ID# NM_001011579.1) was cloned into LakePharma’s proprietary antibiotic selection vector and transfected by electroporation into suspension CHO parental cells. The FLAG-MRJP1-His stable cell line was generated after 2 weeks of antibiotic selection period. Purification of FLAG-MRJP1-His was achieved by a two-step chromatography method. Conditioned media was centrifuged, filtered, and loaded onto an anion exchange chromatography (AEX) resin pre-equilibrated with 20 mM Tris pH 7.5. FLAG-MRJP1-His was eluted by increasing sodium chloride concentration and fractions containing the protein were pooled together. This sample was further polished by a second immobilized metal (Ni) affinity chromatography (IMAC) step using increasing concentrations of imidazole for elution. SDS-PAGE was performed for each fraction and the ones containing FLAG-MRJP1-His were pooled together for dialysis. The final formulation for FLAG-MRJP1-His was 200 mM NaCl in 30 mM HEPES pH 7.0.

For production of supernatants containing recombinant NHLRC3, suspension CHO cells were seeded at 350,000 cells/mL into 90% CD OptiCHO Medium (Thermo Scientific 12681011) containing 6mM L-glutamine (Thermo Scientific 25030–081) and 10% CHO CD EfficientFeed (Thermo Scientific A1023401). CHO cells were grown at 37 °C for 5 days, shaking constantly. The cell suspension was centrifuged at 10,000 × *g* for 40 min and the supernatant run through a 0.22 μm filter. Presence of concentrated NHLRC3 was verified by western blot. The filtered supernatant was concentrated 35-fold and used directly in cell culture assays.

### RNA extraction and quantitative PCR

Total RNA was isolated using TRIzol^®^ (Life Technologies) and RNeasy Kit (QIAGEN) according to the manufacturer’s protocol. cDNA was made with Superscript VILO (Life Technologies). All primers (Supplementary Table 2) were tested for efficiency and single products confirmed. qPCR analyses were performed on the Light Cycler 480II (Roche).

### Lentiviral expression and viral production

pLKO vectors were a gift of Alejandro Sweet-Cordero. N103 vector was a gift of Howard Y. Chang. Sequence verified constructs were used to produce lentivirus using pRSV (Addgene plasmid #12253), pMD2.G (Addgene plasmid #12259), and pMDLg/pRRE (Addgene plasmid #12251). 293Ts were maintained in G418 (Sigma-Aldrich G8168). Plasmids were transfected using Lipofectamine 2000 following manufacturer’s protocol (Thermo-Fisher Scientific 11668). After 12 h, media was changed to viral production media (DMEM, 10% FBS, 1% P/S, 20 mM HEPES). After 48 h, media was collected, spun to remove cell debris, and lentiviral-containing supernatant was added to Lenti-X™ Concentrator (CloneTech). Following incubation at 4 ˚C for >4 h, the mixture was spun at 500 × *g* for 45 min, the pellet resuspended in mESC media, and frozen at −80 ˚C.

### Lentiviral transduction of mESCs

mESCs were plated at a density of 30,000/cm^2^. After 12 h, virus was added with 6 µg/mL polybrene. Media was changed 12 h later and puromycin selection began 48 h post-transduction.

### Cell culture for teratoma formation assay

J1 mESCs were cultured in serum-free media (as described above) with addition of 1000 U/mL mouse LIF, 1 µM PD0325901, and 3 µM CHIR99021, or 0.5 mg/mL Royalactin for three passages. Cells were grown in suspension on Corning Costar Ultra-Low attachment plates (Sigma-Aldrich). Wells were seeded in duplicate at a cell density of 100,000/well, and media and protein were changed daily. To split, colonies were first pelleted by centrifugation at 1500 × *g*, suspended in TrypLE (Life Technologies), incubated at 37 **°**C for 5 min, diluted with PBS (Life Technologies) and pelleted. Cells were counted and re-seeded at a density of 100,000/mL.

### Teratoma generation and histopathology

All animal studies were conducted in accordance with Stanford University animal use guidelines and were approved by the Administrative Panel on Laboratory Animal Care (APLAC). J1 mESCs were mixed with Matrigel (BD 356237) prior to being subcutaneously injected into 8-week-old female SCID/Beige mice (Charles River) on each flank. Four weeks after injection, the mice were euthanized and the teratomas were harvested. All animal studies were approved by Stanford University IACUC guidelines. For histological analysis, slides were stained with hematoxylin and eosin (H&E) following manufacturer’s instructions. Analyses were performed by a board-certified veterinary pathologist.

### Chimera experiments

CGR 8.8 mESCs were grown in serum-free media (as described above) with addition of 1000 U/mL mouse LIF, 1 µM PD0325901, and 3 µM CHIR99021 (2i + LIF), 0i + Royalactin, or 0i + NHLRC3 (as noted) for ten passages. Media was changed daily. Cells were passaged using Accutase (Stemcell Technologies 07920) and suspended in M2 media for injection.

### Protein extraction and western blot analysis

Cellular extracts were prepared using lysis buffer containing 50 mM Tris HCL (pH 7.5), 250 mM NaCl, 1% NP-40, 0.5% Na-deoxycholate, 0.1% SDS, 1 mM phenylmethylsulfonyl fluoride, Halt™ Protease, and Phosphatase Inhibitor Cocktail (ThermoFisher Scientific). Extracts were run on a 4–12% Bis-Tris gel (Novex) and transferred onto PVDF membranes. Blots were blocked in 5% milk PBS-T (TBS for phospho-specific) for 1 h at room temperature followed by overnight incubation at 4 ˚C with primary antibodies. HRP-conjugated secondary antibodies were used at 1:10,000. Antibodies used in this study include Nanog (ReproCELL, RCAB001P, 1:1000), Klf2 (Millipore, 09–280, 1:1000), Esrrb (Perseus Proteomics, PP-H6705–00, 1:500), Stat3 (Cell Signaling, 124H6, 1:1000), pStat3 Y705 (Cell Signaling, D3A7, 1:1000), Sox2 (Santa Cruz, sc-17320, 1:250), Tfcp2l1 (R&D, AF5726, 1:250), NHLRC3 (OriGene, TA336106, 1:500), anti-HA (Cell Signaling, C29F4, 1:1000), and Actin-HRP (Santa Cruz, sc-1616, 1:2500). Uncropped scans of the most important blots are included in Supplementary Figure 5.

### RNA-seq library construction

RNA was extracted using TRIzol and purified on column with the RNeasy Mini Kit (Qiagen). Ribosomal RNA was depleted with the Ribo-Zero Gold rRNA Removal Kit (Illumina). RNA was lyophilized, suspended in 10 μL of water and fragmented to an average size of 200 base pairs using the Ambion RNA Fragmentation Kit (AM8740), and purified using Zymo clean and concentrator 5 columns. 3′ Phosphorylation, adapter ligation, reverse transcription, immunoprecipitation, circularization, amplification, and PAGE separation were performed using the FAST-iCLIP library construction method as previously described^28^. The quality of the libraries, including size distribution and molarity, was assessed on a BioAnalyzer High Sensitivity DNA chip (Agilent). The libraries were then multiplexed and sent for sequencing on an Illumina NextSeq 400 High Output machine for 1 × 75 cycles. Sequencing data deposited under GEO GSE81799.

### ATAC-seq

ATAC-seq was performed on 50,000 J1 mESCs^20^. Cells were grown in serum mESC media (as described above) in serum/+LIF, serum/–LIF, or serum/–LIF + Royalactin for ten passages. Cells were washed with PBS (Life Technologies), trypsinized with Trypsin-EDTA (0.25%), quenched with serum mESC media, washed with PBS, before nuclear isolation with NP-40. Nuclei were resuspended in a transposase reaction mix containing 25µL 2× TD buffer, 2.5 µL Transposase (Illumina) and 22.5 µL of nuclease free water with sequencing adapters. Final libraries were purified on column using the QIAquick PCR Purification Kit (Qiagen) per the manufacturer’s protocol as well as with Agencourt AMPure XP beads (Beckman Coulter) to remove any remaining free adapters. The quality of the libraries, including size distribution and molarity, was assessed on a BioAnalyzer High Sensitivity DNA chip. Libraries were then multiplexed and sent for deep sequencing on the Illumina HiSeq 2500 machine for 2 × 50 cycles. Sequencing data deposited under GEO GSE81799.

### RNA-seq data analysis

Reads were aligned to the mouse reference genome (build mm9) using Tophat. A maximum of a default 2 mismatches was allowed for read alignment. Gene counts were calculated using the HTSeq-count utility^29^ and used as an input for differential gene expression analysis with DESeq version 1.20.0^30^. Genes with a *p*-value of 0.05, as well as a fold change of 2 were selected for further analysis. Validation of top differentially regulated genes was performed with quantitative reverse transcription polymerase chain reaction. Further network analysis on differentially significant genes was performed using NetworkAnalyst^31^. For RNA-seq analysis, GO terms were obtained using DAVID and its default parameters.

PCA analysis was performed using samples as indicated. The genes that led to the maximum amount of variance (PC1) were selected and GO terms obtained using the GO Consortium. Samples from different libraries were normalized using shifted log of normalized counts using DESeq. The ‘plotPCA’ function, which is a part of DESeq2, was used to construct the PCA plots.

### ATAC-seq data analysis

Reads were aligned to the mouse reference genome (build mm9) using Bowtie. The ATAC-seq regions were divided into separate analyses: correlation with closest TSS, correlation with 5356 traditional enhancer regions present in the mm9 genome, and correlation with 361 super-enhancer regions discovered for the mm9 genome^28^. The ATAC-seq signals for serum/+LIF, serum/–LIF, and serum/–LIF + Royalactin after ten passages were compared using DESeq, and the results are represented in the heatmaps. The heatmaps for TSS regions, traditional enhancers, super-enhancers, and differential peaks were produced using unsupervised clustering methods, which used the normalized signal values obtained by quantile normalization, to extract transitions between two states: upregulated and downregulated. The differential peaks between serum/–LIF + Royalactin and serum/–LIF were used for correlation with RNA-seq. The peaks were filtered on the basis of a *p*-value threshold of 0.05 as well as fold change. Boxplots were produced using the ‘BOXPLOT’ function in R. *p*-value was calculated using the student’s *t*-test.

GO terms for peaks differentially expressed in serum/–LIF and serum/–LIF + Royalactin was performed using GREAT. The significant GO terms were filtered to only include GO terms associated with pluripotency and GO terms associated with metabolism. For pluripotency related GO terms, biological processes including morphogenesis, development, proliferation, and stem cell processes were analyzed. For metabolic GO terms, biological processes that were related to metabolism and biosynthetic processes were chosen. Motif analysis for the differential peak lists was performed using HOMER with all differential peaks used as background.

### Structural modeling and Royalactin analog identification

As implemented at the MPI Toolkit (http://toolkit.tuebingen.mpg.de), HHPRED enables sensitive searches of sequence and structural databases through the assembly of profile Hidden Markov Models (HMMs) from a seed sequence, with multiple iterations of Hhblits (a more sensitive and faster program than PSI-BLAST) and PSIPRED (a very accurate secondary structure prediction program)^24^. The detection of a six-bladed β-propeller fold for Royalactin from the top salivary gland protein (PDB ID: 3Q6K) HHPRED hit was accompanied by a significant score of 177.4, an *E*-value of 6e^−28^, and a 28% amino acid identity from the structure-guided overlap of the mature, 413 residue honeybee Royalactin, with the 381 amino acid sand fly SGP. This structural alignment was used by MODELLER^27^ to guide a secure template-guided three-dimensional model of Royalactin (with a VERIFY3D score of 119.7)^32^, and also to nucleate a more sensitive search by HHPRED for a structurally analogous protein (to the greater Royalactin/SGP family) in the human and mouse proteomes. This latter screen, filtered by the signal peptide, single domain, and six-bladed β-propeller fold constraints common to Royalactin and SGPs, yielded NHLRC3 (Uniprot IDs: Q5JS37 and Q8CCH2, for the human and mouse orthologs, respectively) as the sole analog candidate. A three-dimensional model of the NHLRC3 β-propeller was then built by MODELLER on the best six-bladed β-propeller template recognized by HHPRED, Peptidyl-alpha-hydroxyglycine alpha-Amidating Lyase (PDB ID: 3FVZ; at a significant score of 125.6, *E* value of 1.7e-18, and amino acid identity of 24%). The comparison and visualization of Royalactin and NHLRC3 structural models were in turn performed by PyMOL (www.pymol.org). We recognize that β-propeller folds in general (irrespective of ‘blade’ number) are consistently used as interaction scaffolds and preferred binding platforms in the cell^33^. The structural models are available upon request.

## Electronic supplementary material

Supplementary Information

## Data Availability

All relevant data are available from the authors. Sequencing data is also deposited under GEO GSE81799.
