# Supplementary material for: Honey bee Royalactin unlocks conserved pluripotency pathway in mammals
Source: Nat Commun. 2018 Dec 4;9:5078. doi: 10.1038/s41467-018-06256-4 (PMC6277453; doi:10.1038/s41467-018-06256-4)
Supplement: Supplementary file 1 — Supplementary Information [file 41467_2018_6256_MOESM1_ESM.pdf]

Supplementary Information

Honey Bee Royalactin Unlocks Conserved Pluripotency Pathway in Mammals

Wan et al.

Supplementary Fig 1

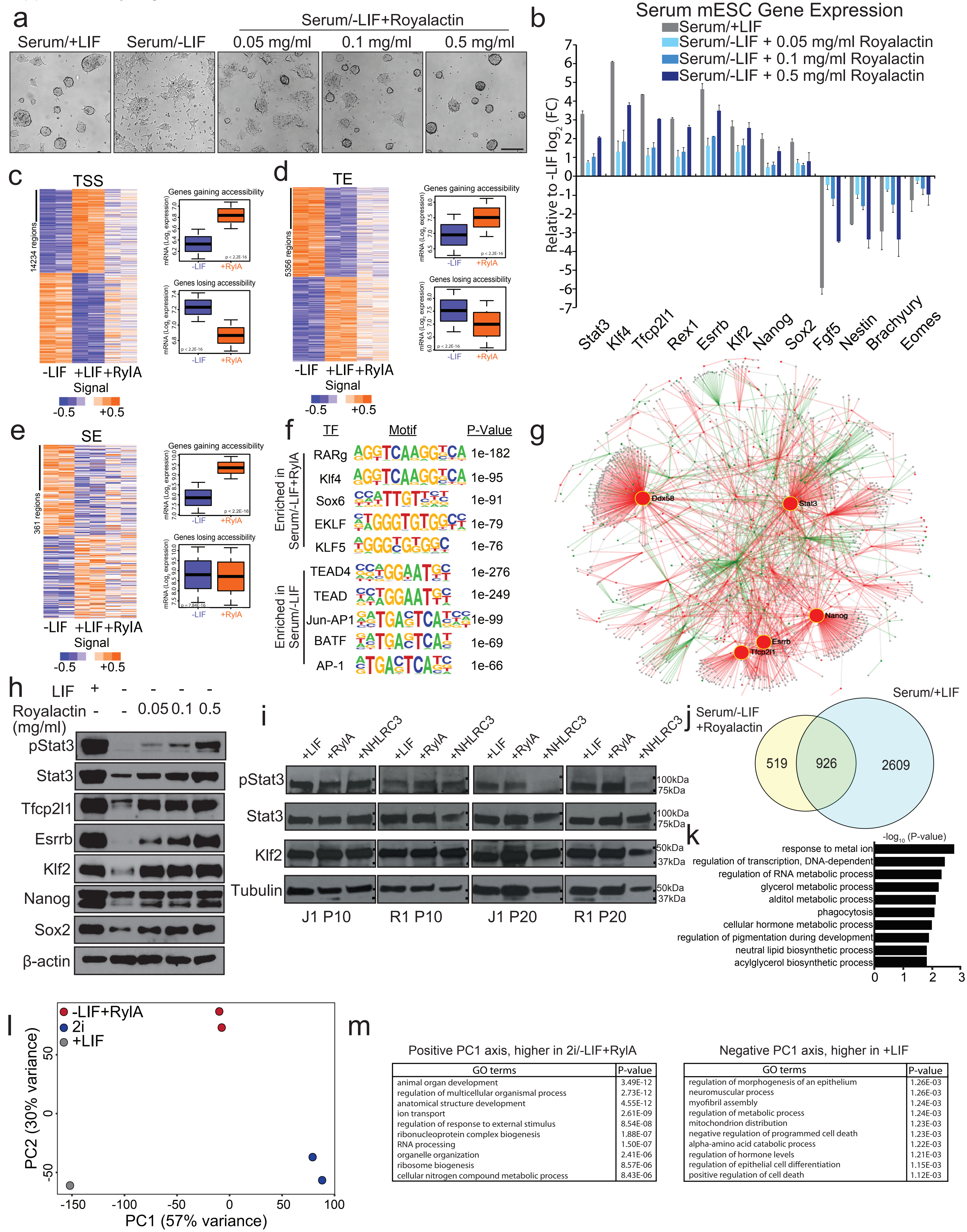

Positive PC1 axis, higher in 2i/-LIF+RylA

GO terms	P-value
animal organ development	3.49E-12
regulation of multicellular organismal process	2.73E-12
anatomical structure development	4.55E-12
ion transport	2.61E-09
regulation of response to external stimulus	8.54E-08
ribonucleoprotein complex biogenesis	1.88E-07
RNA processing	1.50E-07
organelle organization	2.41E-06
ribosome biogenesis	8.57E-06
cellular nitrogen compound metabolic process	8.43E-06

Negative PC1 axis, higher in +LIF

GO terms	P-value
regulation of morphogenesis of an epithelium	1.26E-03
neuromuscular process	1.26E-03
myofibril assembly	1.24E-03
regulation of metabolic process	1.24E-03
mitochondrion distribution	1.23E-03
negative regulation of programmed cell death	1.23E-03
alpha-amino acid catabolic process	1.22E-03
regulation of hormone levels	1.21E-03
regulation of epithelial cell differentiation	1.15E-03
positive regulation of cell death	1.12E-03

Supplementary Fig. 1 Royalactin confers self-renewal and activates the pluripotent gene regulatory network.

(a) Representative images of J1 mESCs cultured in serum/+LIF, serum/-LIF, or serum/-LIF+Royalactin 3 passages. After LIF withdrawal, mESCs rapidly differentiated, whereas cells cultured with Royalactin demonstrated dose-dependent response supporting self-renewal with negligible differentiation. Scale bar, 200 µm.
(b) Quantitative expression of pluripotency and differentiation-associated genes from (a). Data are means ± SD (n=2).
(c) Left: ATAC-seq activity in TSS regions of J1 mESCs in response to serum/+LIF (+LIF), serum/-LIF+Royalactin (+RylA), and serum/-LIF (-LIF) after 10 passages. Each column is a sample, each row is an element. Samples and elements organized by unsupervised *k*-means clustering. Right: boxplots of mRNA expression levels of indicated genes in serum/-LIF or serum/-LIF+Royalactin cells. *p*-value estimated from Student's t-test.
(d) Heatmap and boxplots as in (c) for ATAC-seq activity in traditional enhancer (TE) regions of J1 mESCs in response to serum/+LIF (+LIF), serum/-LIF+Royalactin (+RylA), and serum/-LIF (-LIF) after 10 passages.
(e) Heatmap and boxplots as in (c) for ATAC-seq activity in super-enhancer (SE) regions of J1 mESCs in response to serum/+LIF (+LIF), serum/-LIF+Royalactin (+RylA), and serum/-LIF (-LIF) after 10 passages.
(f) Motif analysis of royalactin-upregulated SE constituents from (e).
(g) Network analysis of differentially expressed genes in J1 mESCs cultured in serum/-LIF+Royalactin relative to serum/-LIF for 10 passages.
(h) Representative Western blot analysis demonstrating dose-dependent response of main factors implicated in maintenance of pluripotency to Royalactin in J1 mESCs cultured in serum/+LIF (+LIF) and increasing concentrations of serum/-LIF+Royalactin (+RylA) for 3 passages.
(i) Representative Western blot analysis of factors implicated in maintenance of pluripotency in J1 and R1 mESCs in serum/+LIF (+LIF), serum/-LIF+Royalactin (+RylA), and serum/-LIF+NHLRC3 (+NHLRC3) after 10 and 20 passages.
(j) Overlap between genes upregulated in serum/-LIF+Royalactin and serum/+LIF (both compared to serum/-LIF) after 3 passages.
(k) Associated GO terms for 519 serum/-LIF+Royalactin specific genes from (j).
(l) J1 mESCs cultured in serum/+LIF, serum/-LIF+Royalactin, and 2i+LIF projected onto first two principal components. All genes with mean normalized read counts larger than 10 were considered for principal component analysis (PCA).
(m) Distribution of genes contributing to principal component 1 (PC1) in (l), with GO term enrichment analysis of genes most strongly contributing to PC1 separation.

Supplementary Figure 2

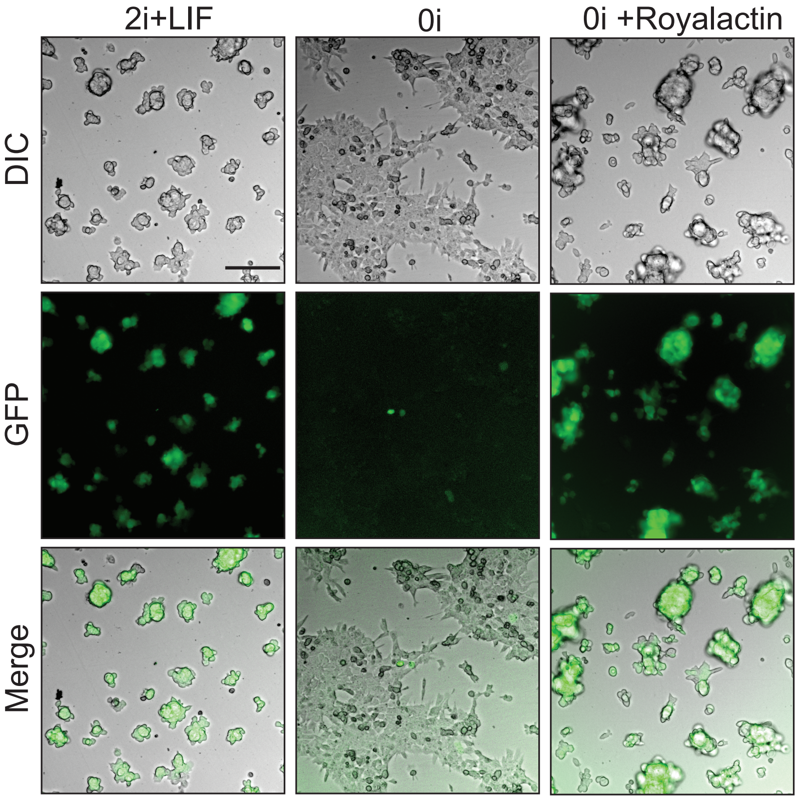

Supplementary Fig. 2 Royalactin maintains pluripotency in mESCs in the absence of serum. Rex1-GFP reporter mESCs demonstrated maintenance of pluripotency in cells cultured in 2i+LIF, whereas cells cultured in 0i differentiated. However, addition of Royalactin (0i+Royalactin) maintained pluripotency for over 10 passages. Scale bar, 200 μm.

Supplementary Figure 3

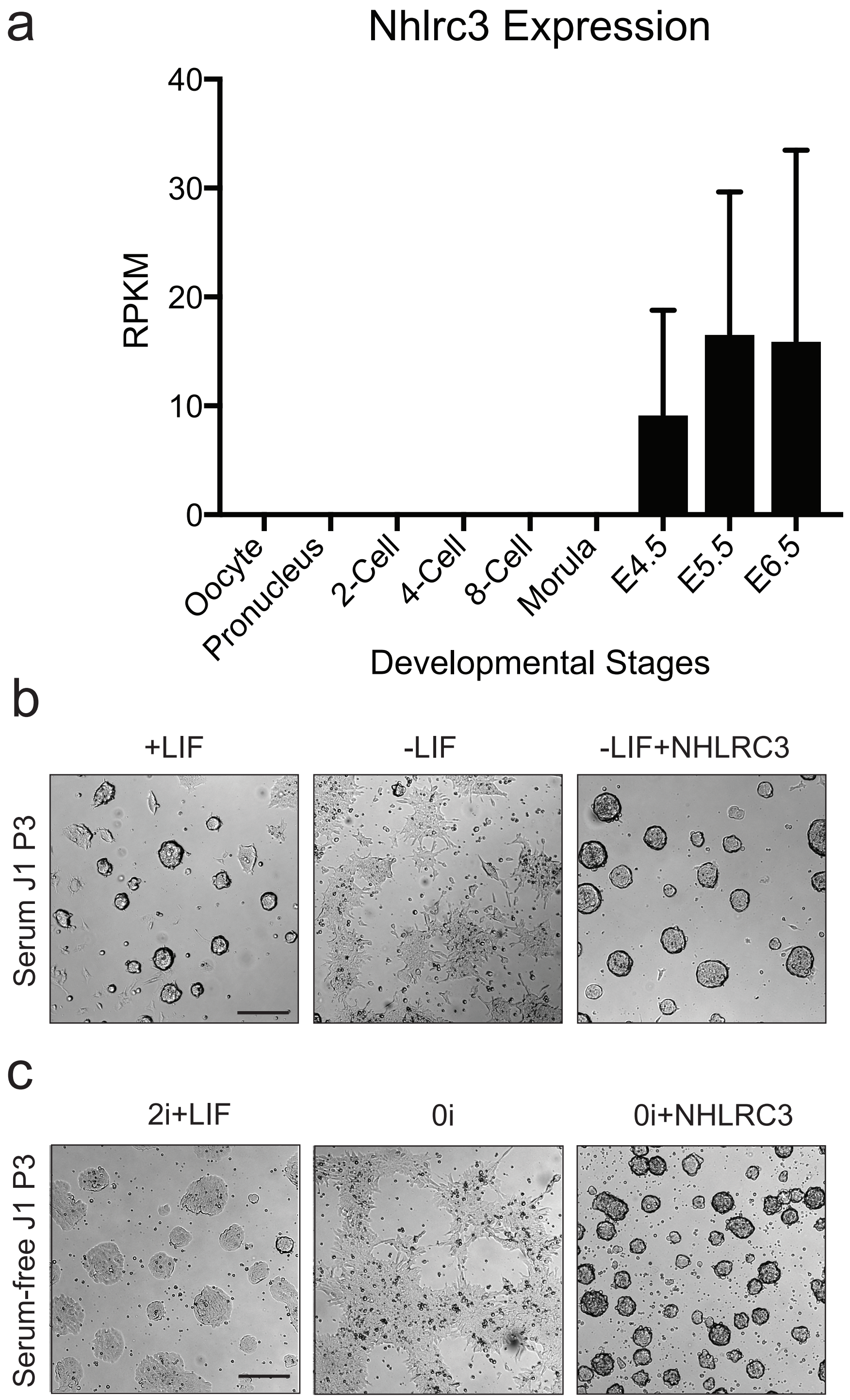

Supplementary Fig. 3 NHLRC3 is expressed in murine embryos and maintains pluripotent morphology in mESCs in the presence or absence of serum.

(a) RNA-seq of single embryos demonstrates that Nhlrc3 begins to be expressed at E4.5 and increases steadily thereafter.

(b) Additional representative images of J1 mESCs cultured in serum/+LIF, serum/-LIF, or serum/-LIF+NHLRC3 for 3 passages. After LIF withdrawal, mESCs rapidly differentiated, whereas cells cultured with NHLRC3 supported self-renewal with negligible differentiation. Scale bar, 200 μm.

(c) Additional representative images of J1 mESCs cultured in serum-free media in the presence (2i+LIF) or absence (0i) of MAPKKi, GSK3i, and LIF for 3 passages. mESCs rapidly differentiated in 0i, whereas cells cultured with NHLRC3 (0i+NHLRC3) supported self-renewal with negligible differentiation. Scale bar, 200 μm.

Supplementary Figure 4

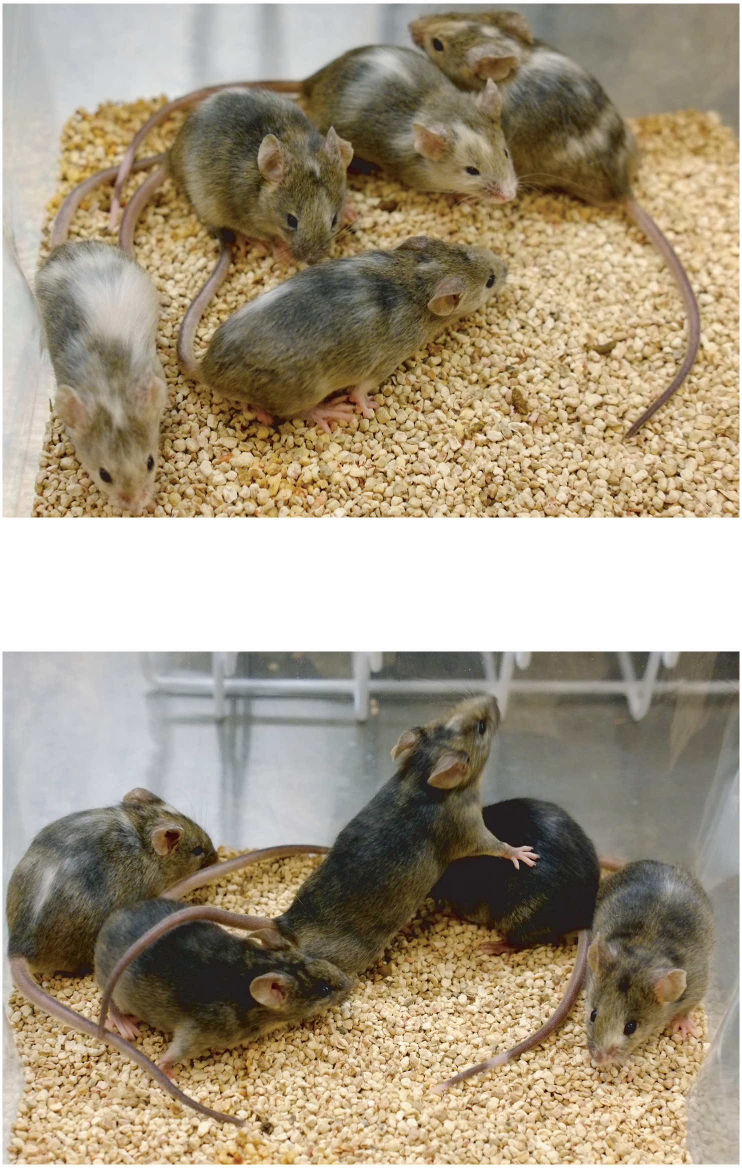

Supplementary Fig. 4 NHLRC3 cultured mESCs self-renew and generate chimeric animals. Representative photographs of chimeric animals generated from CGR 8.8 mESCs cultured in 0i/-LIF+NHLRC3 for 10 passages and injected into mouse blastocysts, as demonstrated by coat-color chimerism.

Supplementary Figure 5

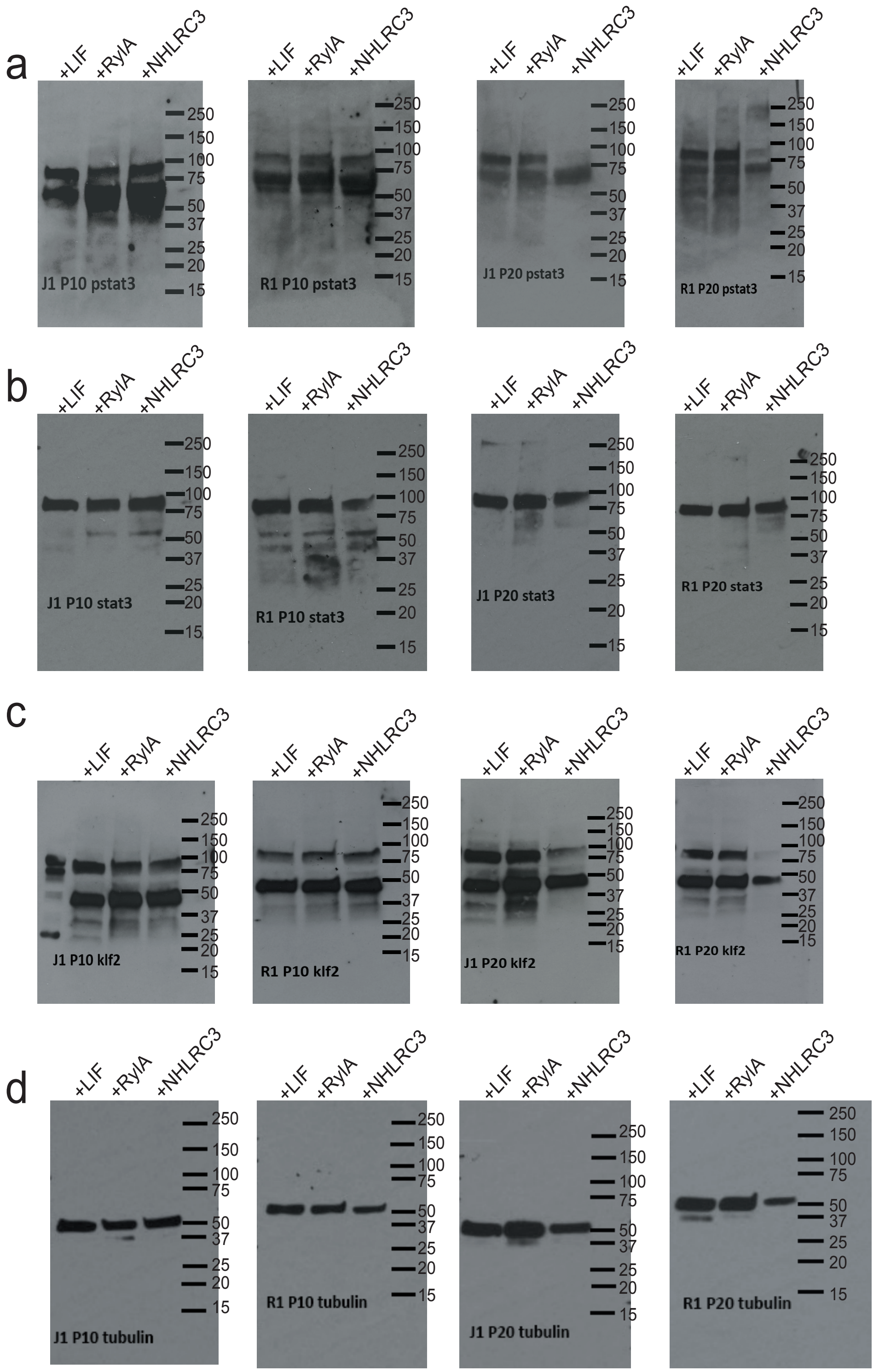

Supplementary Fig. 5 Uncropped images of western blots shown in Supplementary Figure 1i. Original scans of western blots for pStat3 (a), Stat3 (b), Klf2 (c), and Tubulin (d) as shown in Supplementary Figure 1i.

Supplementary Table 1. Assessment of Chimeras

Cohort 1 – Figure 2C Left:

- 5 females
  - 2 chimeras: 55%, 40%
  - 3 non-chimeras

Cohort 2 – Figure 2C Right:

- 3 males
  - 3 chimeras: 40%, 20%, 5%

Cohort 3 – Supplementary Figure 4 Top:

- 5 males
  - 5 chimeras: all 90%

Cohort 4 – Supplementary Figure 4 Bottom:

- 5 males
  - 5 chimeras: 90%, 90%, 60%, 60%, 40%

Additional chimeras (images not shown):

Cohort 5 – Royalactin:

- 2 males, 3 females
  - 2 male chimeras: 60%, 20%
  - 2 female chimeras: 60%, 20%
  - 1 female non-chimera

Cohort 6 – NHLRC3:

- 5 females
  - 3 chimeras: 60%, 60%, 40%
  - 2 non-chimeras

Supplementary Table 2. List of qPCR primers and shRNA sequences. Table of shRNAs with their TRC ID numbers, qPCR primers. qPCR primers were verified for efficiency and expected, single product size confirmed on a DNA agarose gel.

shRNA Sequences

Gene Name	cloneId	shRNA target sequence
Scramble		CAACAAGATGAAGAGCACCAA
Stat3	TRCN0000071454	CGACTTTGATTTCAACTACAA
Esrrb	TRCN0000026168	GCGCAGGTACAAGAAACTCAA
Tfcp2l1	TRCN0000082074	CGGCTCAAGAGAAGGAGAAAT
Klf2	TRCN0000081905	CCTAAACAACGTGTTGGACTT
EGFR	TRCN0000055220	CCAAGCCAAATGGCATATTTA
LIFR	TRCN0000065613	GCAGAGATACAGCTTAGTAAA
gp130	TRCN0000065390	CCTCCAGATAAACCTACAAAT

mESC qPCR Primers

Gene Name	Forward qPCR primer	Reverse qPCR primer
Stat3	GCCACGTTGGTGTTTCATAA	AGACTCTTCCCACAGGCATC
Klf4	GTGCAGCTTGCAGCAGTAAC	AGCGAGTTGGAAAGGATAA
Tfcp2l1	AGCTGCCAGATCAAGGTGTT	TCGGTAAGGATGGTGGTTTC
Socs3	CTCCAGGGACCCCCTCCTTTTCTT	CAAAGATGCTGGAGGGTGGCCA
Esrrb	GACATTGCCTCTGGCTACCACT	ACTTGCGCCTCCGTTTGGTGAT
Klf2	CCAAGAGCTCGCACCTAAAG	AGTGGCACTGAAAGGGTCTG
Nanog	CCTCAGCCTCCAGCAGATGC	CCGCTTGCACTTCATCCTTTG
Sox2	CTGGTCATGGAGTTGTACTGCAGG	GGCAGCTACAGCATGATGCAGGAGC
Oct4	GCTCACCCTGGGCGTTCTC	GGCCGCAGCTTACACATGTTC
Rex1	TTCACGGAGAGCTCGAAACT	CTTTGCGTGGGTTAGGATGT
Fgf5	GAAAAGACAGGCCGAGAGTG	GAAGTGGGTGGAGACGTGTT
Nestin	CCAGAGCTGGACTGGAACTC	ACCTGCCTCTTTTGGTTCCT
Brachyury	CCGGTGCTGAAGGTAAATGT	CCTCCATTGAGCTTGTTGGT
Eomes	ACCCAGCTAAAGATCGACCA	GAAGTTTTGAACGCCGTACC
EGFR	TTGGCCTATTCATGCGAAGAC	GAGGTTCCACGAGCTCTCTCTCT
LIFR	GCCTCATTTCTCCGGTTACA	GTTTGCCCGTACAGATGGAT
gp130	TCATGTTCCTTCTATCGGGTC	CTGAGGGACCGGTGGTGT
Ppib	GTGAGCGCTTCCCAGATGAGA	TGCCGGAGTCGACAATGATG
